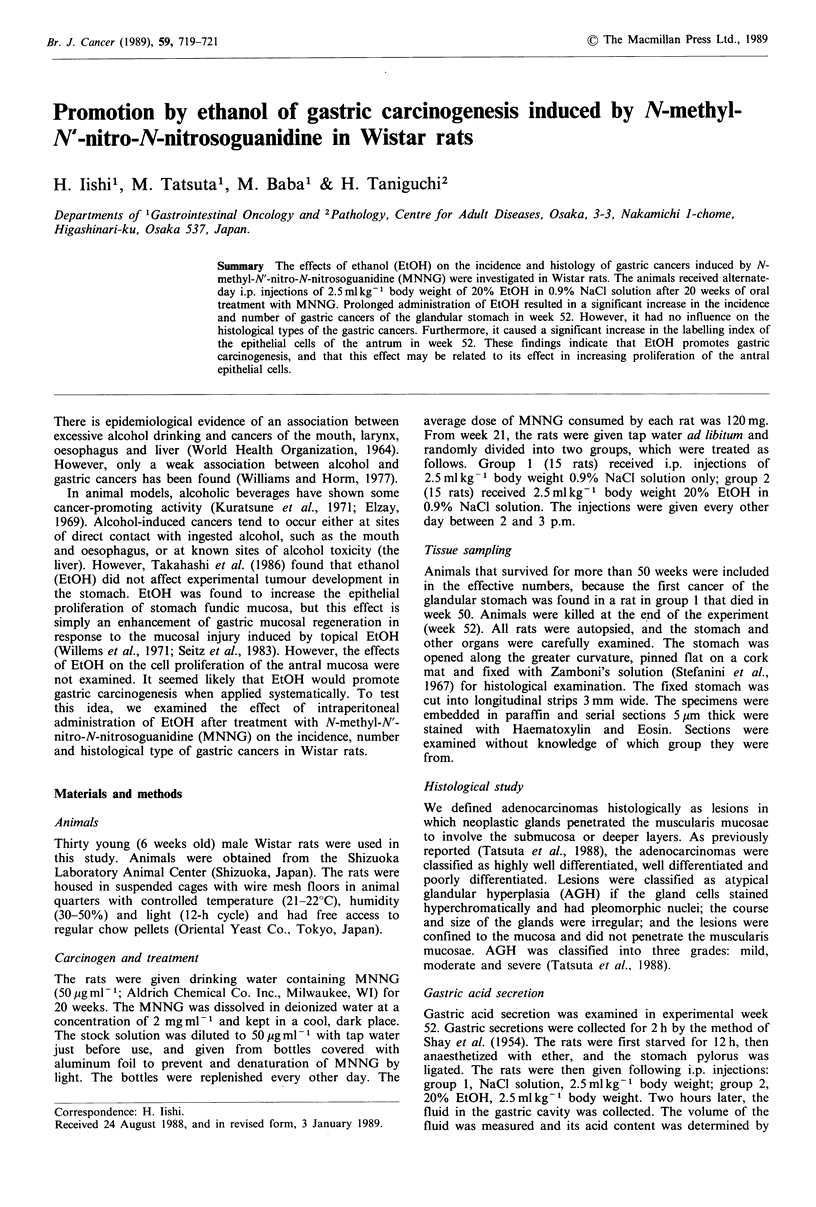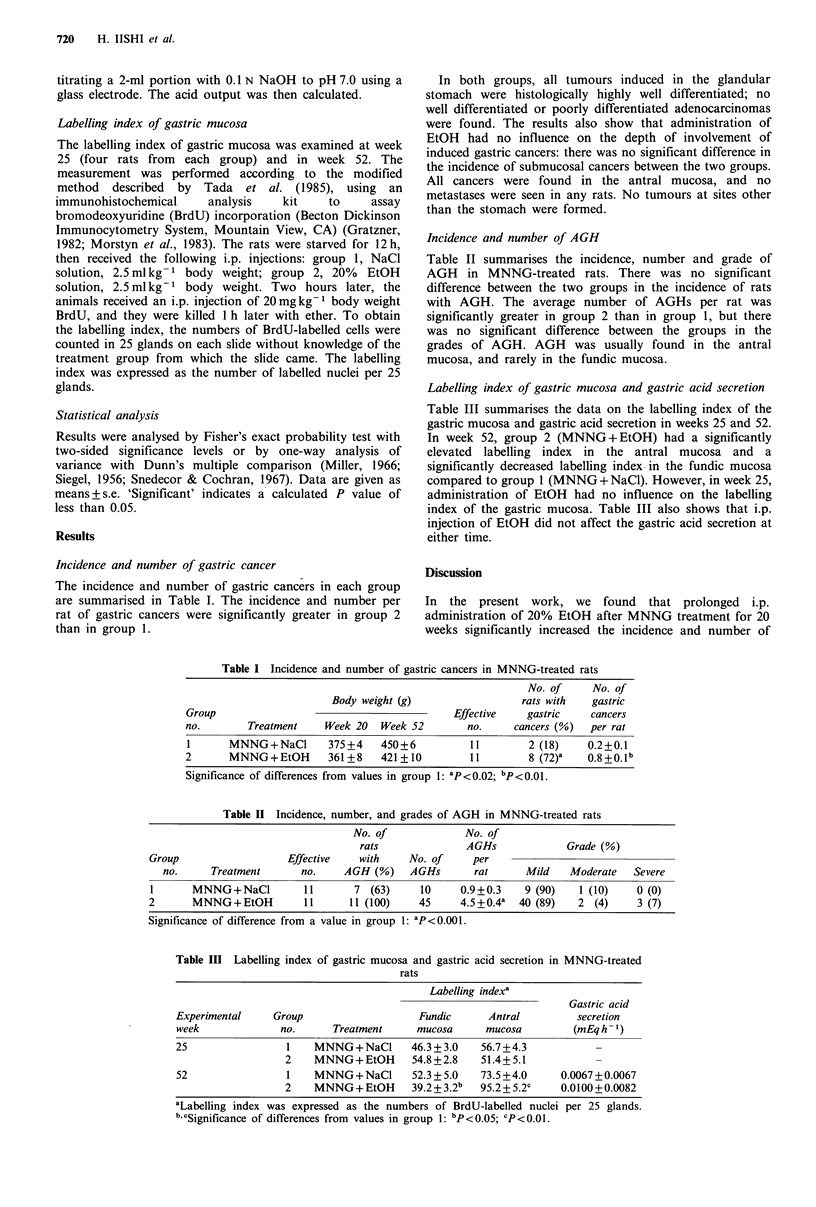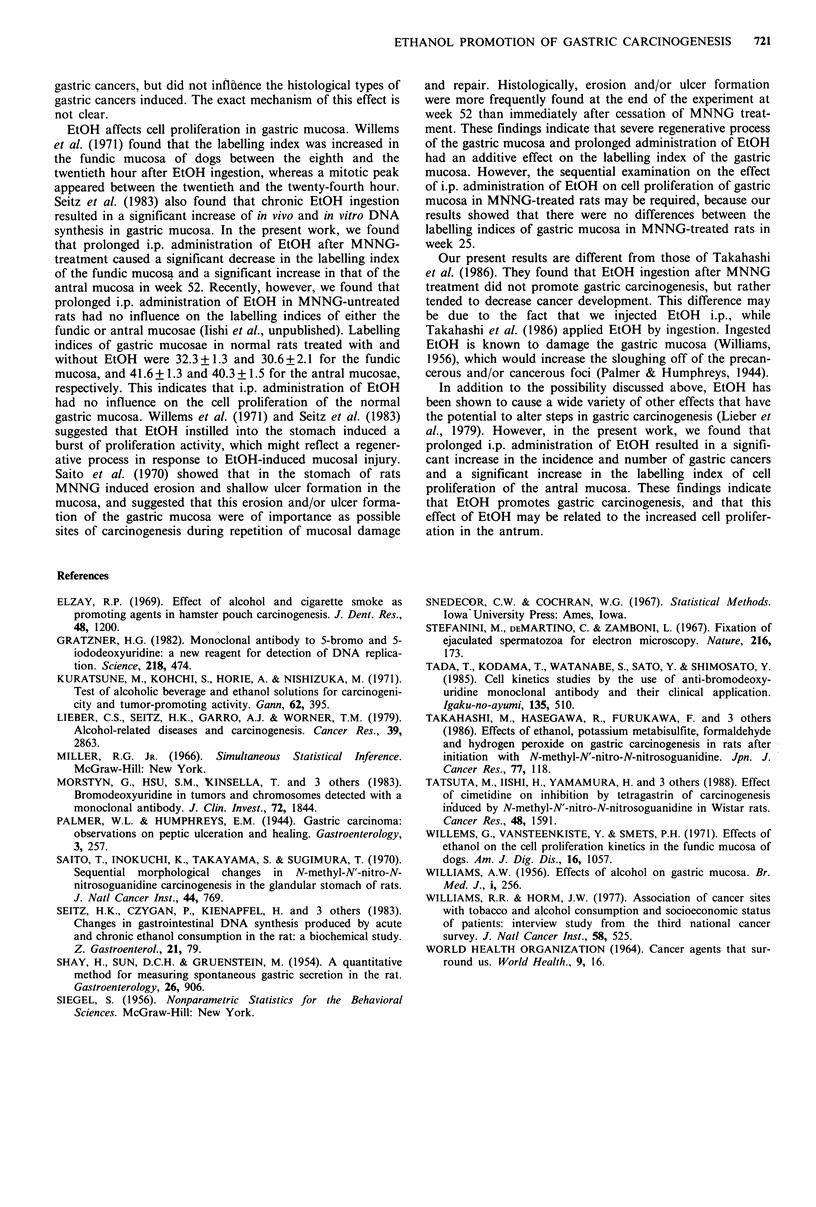# Promotion by ethanol of gastric carcinogenesis induced by N-methyl-N'-nitro-N-nitrosoguanidine in Wistar rats.

**DOI:** 10.1038/bjc.1989.151

**Published:** 1989-05

**Authors:** H. Iishi, M. Tatsuta, M. Baba, H. Taniguchi

**Affiliations:** Department of Gastrointestinal Oncology, Centre for Adult Diseases, Osaka, Japan.

## Abstract

The effects of ethanol (EtOH) on the incidence and histology of gastric cancers induced by N-methyl-N'-nitro-N-nitrosoguanidine (MNNG) were investigated in Wistar rats. The animals received alternate-day i.p. injections of 2.5 ml kg-1 body weight of 20% EtOH in 0.9% NaCl solution after 20 weeks of oral treatment with MNNG. Prolonged administration of EtOH resulted in a significant increase in the incidence and number of gastric cancers of the glandular stomach in week 52. However, it had no influence on the histological types of the gastric cancers. Furthermore, it caused a significant increase in the labelling index of the epithelial cells of the antrum in week 52. These findings indicate that EtOH promotes gastric carcinogenesis, and that this effect may be related to its effect in increasing proliferation of the antral epithelial cells.


*Br. J. Cancer* (1989), **59**, 719–721 © The Macmillan Press Ltd., 1989

### Promotion by ethanol of gastric carcinogenesis induced by *N*-methyl-*N'*-nitro-*N*-nitrosoguanidine in Wistar rats

H. Iishi[1], M. Tatsuta[1], M. Baba[1] & H. Taniguchi[2]

*Departments of [1]Gastrointestinal Oncology and [2]Pathology, Centre for Adult Diseases, Osaka, 3-3, Nakamichi 1-chome, Higashinari-ku, Osaka 537, Japan.*

**Summary** The effects of ethanol (EtOH) on the incidence and histology of gastric cancers induced by *N*-methyl-*N'*-nitro-*N*-nitrosoguanidine (MNNG) were investigated in Wistar rats. The animals received alternate-day i.p. injections of 2.5 ml $kg^{-1}$ body weight of 20% EtOH in 0.9% NaCl solution after 20 weeks of oral treatment with MNNG. Prolonged administration of EtOH resulted in a significant increase in the incidence and number of gastric cancers of the glandular stomach in week 52. However, it had no influence on the histological types of the gastric cancers. Furthermore, it caused a significant increase in the labelling index of the epithelial cells of the antrum in week 52. These findings indicate that EtOH promotes gastric carcinogenesis, and that this effect may be related to its effect in increasing proliferation of the antral epithelial cells.

There is epidemiological evidence of an association between excessive alcohol drinking and cancers of the mouth, larynx, oesophagus and liver (World Health Organization, 1964). However, only a weak association between alcohol and gastric cancers has been found (Williams and Horm, 1977).

In animal models, alcoholic beverages have shown some cancer-promoting activity (Kuratsune *et al.*, 1971; Elzay, 1969). Alcohol-induced cancers tend to occur either at sites of direct contact with ingested alcohol, such as the mouth and oesophagus, or at known sites of alcohol toxicity (the liver). However, Takahashi *et al.* (1986) found that ethanol (EtOH) did not affect experimental tumour development in the stomach. EtOH was found to increase the epithelial proliferation of stomach fundic mucosa, but this effect is simply an enhancement of gastric mucosal regeneration in response to the mucosal injury induced by topical EtOH (Willems *et al.*, 1971; Seitz *et al.*, 1983). However, the effects of EtOH on the cell proliferation of the antral mucosa were not examined. It seemed likely that EtOH would promote gastric carcinogenesis when applied systematically. To test this idea, we examined the effect of intraperitoneal administration of EtOH after treatment with *N*-methyl-*N'*-nitro-*N*-nitrosoguanidine (MNNG) on the incidence, number and histological type of gastric cancers in Wistar rats.

### Materials and methods

### *Animals*

Thirty young (6 weeks old) male Wistar rats were used in this study. Animals were obtained from the Shizuoka Laboratory Animal Center (Shizuoka, Japan). The rats were housed in suspended cages with wire mesh floors in animal quarters with controlled temperature (21–22°C), humidity (30–50%) and light (12-h cycle) and had free access to regular chow pellets (Oriental Yeast Co., Tokyo, Japan).

### *Carcinogen and treatment*

The rats were given drinking water containing MNNG (50 $\mu g\,ml^{-1}$; Aldrich Chemical Co. Inc., Milwaukee, WI) for 20 weeks. The MNNG was dissolved in deionized water at a concentration of 2 mg $ml^{-1}$ and kept in a cool, dark place. The stock solution was diluted to 50 $\mu g\,ml^{-1}$ with tap water just before use, and given from bottles covered with aluminum foil to prevent and denaturation of MNNG by light. The bottles were replenished every other day. The average dose of MNNG consumed by each rat was 120 mg. From week 21, the rats were given tap water *ad libitum* and randomly divided into two groups, which were treated as follows. Group 1 (15 rats) received i.p. injections of 2.5 ml $kg^{-1}$ body weight 0.9% NaCl solution only; group 2 (15 rats) received 2.5 ml $kg^{-1}$ body weight 20% EtOH in 0.9% NaCl solution. The injections were given every other day between 2 and 3 p.m.

Correspondence: H. Iishi.
Received 24 August 1988, and in revised form, 3 January 1989.

### *Tissue sampling*

Animals that survived for more than 50 weeks were included in the effective numbers, because the first cancer of the glandular stomach was found in a rat in group 1 that died in week 50. Animals were killed at the end of the experiment (week 52). All rats were autopsied, and the stomach and other organs were carefully examined. The stomach was opened along the greater curvature, pinned flat on a cork mat and fixed with Zamboni's solution (Stefanini *et al.*, 1967) for histological examination. The fixed stomach was cut into longitudinal strips 3 mm wide. The specimens were embedded in paraffin and serial sections 5 $\mu m$ thick were stained with Haematoxylin and Eosin. Sections were examined without knowledge of which group they were from.

### *Histological study*

We defined adenocarcinomas histologically as lesions in which neoplastic glands penetrated the muscularis mucosae to involve the submucosa or deeper layers. As previously reported (Tatsuta *et al.*, 1988), the adenocarcinomas were classified as highly well differentiated, well differentiated and poorly differentiated. Lesions were classified as atypical glandular hyperplasia (AGH) if the gland cells stained hyperchromatically and had pleomorphic nuclei; the course and size of the glands were irregular; and the lesions were confined to the mucosa and did not penetrate the muscularis mucosae. AGH was classified into three grades: mild, moderate and severe (Tatsuta *et al.*, 1988).

### *Gastric acid secretion*

Gastric acid secretion was examined in experimental week 52. Gastric secretions were collected for 2 h by the method of Shay *et al.* (1954). The rats were first starved for 12 h, then anaesthetized with ether, and the stomach pylorus was ligated. The rats were then given following i.p. injections: group 1, NaCl solution, 2.5 ml $kg^{-1}$ body weight; group 2, 20% EtOH, 2.5 ml $kg^{-1}$ body weight. Two hours later, the fluid in the gastric cavity was collected. The volume of the fluid was measured and its acid content was determined by titrating a 2-ml portion with 0.1 N NaOH to pH 7.0 using a glass electrode. The acid output was then calculated.

*Labelling index of gastric mucosa*

The labelling index of gastric mucosa was examined at week 25 (four rats from each group) and in week 52. The measurement was performed according to the modified method described by Tada *et al.* (1985), using an immunohistochemical analysis kit to assay bromodeoxyuridine (BrdU) incorporation (Becton Dickinson Immunocytometry System, Mountain View, CA) (Gratzner, 1982; Morstyn *et al.*, 1983). The rats were starved for 12 h, then received the following i.p. injections: group 1, NaCl solution, $2.5\,ml\,kg^{-1}$ body weight; group 2, 20% EtOH solution, $2.5\,ml\,kg^{-1}$ body weight. Two hours later, the animals received an i.p. injection of $20\,mg\,kg^{-1}$ body weight BrdU, and they were killed 1 h later with ether. To obtain the labelling index, the numbers of BrdU-labelled cells were counted in 25 glands on each slide without knowledge of the treatment group from which the slide came. The labelling index was expressed as the number of labelled nuclei per 25 glands.

*Statistical analysis*

Results were analysed by Fisher's exact probability test with two-sided significance levels or by one-way analysis of variance with Dunn's multiple comparison (Miller, 1966; Siegel, 1956; Snedecor & Cochran, 1967). Data are given as means ± s.e. 'Significant' indicates a calculated $P$ value of less than 0.05.

### Results

*Incidence and number of gastric cancer*

The incidence and number of gastric cancers in each group are summarised in Table I. The incidence and number per rat of gastric cancers were significantly greater in group 2 than in group 1.

In both groups, all tumours induced in the glandular stomach were histologically highly well differentiated; no well differentiated or poorly differentiated adenocarcinomas were found. The results also show that administration of EtOH had no influence on the depth of involvement of induced gastric cancers: there was no significant difference in the incidence of submucosal cancers between the two groups. All cancers were found in the antral mucosa, and no metastases were seen in any rats. No tumours at sites other than the stomach were formed.

*Incidence and number of AGH*

Table II summarises the incidence, number and grade of AGH in MNNG-treated rats. There was no significant difference between the two groups in the incidence of rats with AGH. The average number of AGHs per rat was significantly greater in group 2 than in group 1, but there was no significant difference between the groups in the grades of AGH. AGH was usually found in the antral mucosa, and rarely in the fundic mucosa.

*Labelling index of gastric mucosa and gastric acid secretion*

Table III summarises the data on the labelling index of the gastric mucosa and gastric acid secretion in weeks 25 and 52. In week 52, group 2 (MNNG + EtOH) had a significantly elevated labelling index in the antral mucosa and a significantly decreased labelling index in the fundic mucosa compared to group 1 (MNNG + NaCl). However, in week 25, administration of EtOH had no influence on the labelling index of the gastric mucosa. Table III also shows that i.p. injection of EtOH did not affect the gastric acid secretion at either time.

### Discussion

In the present work, we found that prolonged i.p. administration of 20% EtOH after MNNG treatment for 20 weeks significantly increased the incidence and number of

**Table I** Incidence and number of gastric cancers in MNNG-treated rats

| Group no. | Treatment | Body weight (g) Week 20 | Week 52 | Effective no. | No. of rats with gastric cancers (%) | No. of gastric cancers per rat |
|---|---|---|---|---|---|---|
| 1 | MNNG + NaCl | 375 ± 4 | 450 ± 6 | 11 | 2 (18) | 0.2 ± 0.1 |
| 2 | MNNG + EtOH | 361 ± 8 | 421 ± 10 | 11 | 8 (72)[a] | 0.8 ± 0.1[b] |

Significance of differences from values in group 1: [a]$P<0.02$; [b]$P<0.01$.

**Table II** Incidence, number, and grades of AGH in MNNG-treated rats

| Group no. | Treatment | Effective no. | No. of rats with AGH (%) | No. of AGHs | No. of AGHs per rat | Grade (%) Mild | Moderate | Severe |
|---|---|---|---|---|---|---|---|---|
| 1 | MNNG + NaCl | 11 | 7 (63) | 10 | 0.9 ± 0.3 | 9 (90) | 1 (10) | 0 (0) |
| 2 | MNNG + EtOH | 11 | 11 (100) | 45 | 4.5 ± 0.4[a] | 40 (89) | 2 (4) | 3 (7) |

Significance of difference from a value in group 1: [a]$P<0.001$.

**Table III** Labelling index of gastric mucosa and gastric acid secretion in MNNG-treated rats

| Experimental week | Group no. | Treatment | Labelling index[a] Fundic mucosa | Antral mucosa | Gastric acid secretion ($mEq\,h^{-1}$) |
|---|---|---|---|---|---|
| 25 | 1 | MNNG + NaCl | 46.3 ± 3.0 | 56.7 ± 4.3 | – |
| | 2 | MNNG + EtOH | 54.8 ± 2.8 | 51.4 ± 5.1 | – |
| 52 | 1 | MNNG + NaCl | 52.3 ± 5.0 | 73.5 ± 4.0 | 0.0067 ± 0.0067 |
| | 2 | MNNG + EtOH | 39.2 ± 3.2[b] | 95.2 ± 5.2[c] | 0.0100 ± 0.0082 |

[a]Labelling index was expressed as the numbers of BrdU-labelled nuclei per 25 glands. [b,c]Significance of differences from values in group 1: [b]$P<0.05$; [c]$P<0.01$.

gastric cancers, but did not influence the histological types of gastric cancers induced. The exact mechanism of this effect is not clear.

EtOH affects cell proliferation in gastric mucosa. Willems *et al.* (1971) found that the labelling index was increased in the fundic mucosa of dogs between the eighth and the twentieth hour after EtOH ingestion, whereas a mitotic peak appeared between the twentieth and the twenty-fourth hour. Seitz *et al.* (1983) also found that chronic EtOH ingestion resulted in a significant increase of *in vivo* and *in vitro* DNA synthesis in gastric mucosa. In the present work, we found that prolonged i.p. administration of EtOH after MNNG-treatment caused a significant decrease in the labelling index of the fundic mucosa and a significant increase in that of the antral mucosa in week 52. Recently, however, we found that prolonged i.p. administration of EtOH in MNNG-untreated rats had no influence on the labelling indices of either the fundic or antral mucosae (Iishi *et al.*, unpublished). Labelling indices of gastric mucosae in normal rats treated with and without EtOH were $32.3 \pm 1.3$ and $30.6 \pm 2.1$ for the fundic mucosa, and $41.6 \pm 1.3$ and $40.3 \pm 1.5$ for the antral mucosae, respectively. This indicates that i.p. administration of EtOH had no influence on the cell proliferation of the normal gastric mucosa. Willems *et al.* (1971) and Seitz *et al.* (1983) suggested that EtOH instilled into the stomach induced a burst of proliferation activity, which might reflect a regenerative process in response to EtOH-induced mucosal injury. Saito *et al.* (1970) showed that in the stomach of rats MNNG induced erosion and shallow ulcer formation in the mucosa, and suggested that this erosion and/or ulcer formation of the gastric mucosa were of importance as possible sites of carcinogenesis during repetition of mucosal damage and repair. Histologically, erosion and/or ulcer formation were more frequently found at the end of the experiment at week 52 than immediately after cessation of MNNG treatment. These findings indicate that severe regenerative process of the gastric mucosa and prolonged administration of EtOH had an additive effect on the labelling index of the gastric mucosa. However, the sequential examination on the effect of i.p. administration of EtOH on cell proliferation of gastric mucosa in MNNG-treated rats may be required, because our results showed that there were no differences between the labelling indices of gastric mucosa in MNNG-treated rats in week 25.

Our present results are different from those of Takahashi *et al.* (1986). They found that EtOH ingestion after MNNG treatment did not promote gastric carcinogenesis, but rather tended to decrease cancer development. This difference may be due to the fact that we injected EtOH i.p., while Takahashi *et al.* (1986) applied EtOH by ingestion. Ingested EtOH is known to damage the gastric mucosa (Williams, 1956), which would increase the sloughing off of the precancerous and/or cancerous foci (Palmer & Humphreys, 1944).

In addition to the possibility discussed above, EtOH has been shown to cause a wide variety of other effects that have the potential to alter steps in gastric carcinogenesis (Lieber *et al.*, 1979). However, in the present work, we found that prolonged i.p. administration of EtOH resulted in a significant increase in the incidence and number of gastric cancers and a significant increase in the labelling index of cell proliferation of the antral mucosa. These findings indicate that EtOH promotes gastric carcinogenesis, and that this effect of EtOH may be related to the increased cell proliferation in the antrum.

### References

ELZAY, R.P. (1969). Effect of alcohol and cigarette smoke as promoting agents in hamster pouch carcinogenesis. *J. Dent. Res.*, **48**, 1200.

GRATZNER, H.G. (1982). Monoclonal antibody to 5-bromo and 5-iododeoxyuridine: a new reagent for detection of DNA replication. *Science*, **218**, 474.

KURATSUNE, M., KOHCHI, S., HORIE, A. & NISHIZUKA, M. (1971). Test of alcoholic beverage and ethanol solutions for carcinogenicity and tumor-promoting activity. *Gann*, **62**, 395.

LIEBER, C.S., SEITZ, H.K., GARRO, A.J. & WORNER, T.M. (1979). Alcohol-related diseases and carcinogenesis. *Cancer Res.*, **39**, 2863.

MILLER, R.G. JR. (1966). *Simultaneous Statistical Inference*. McGraw-Hill: New York.

MORSTYN, G., HSU, S.M., KINSELLA, T. and 3 others (1983). Bromodeoxyuridine in tumors and chromosomes detected with a monoclonal antibody. *J. Clin. Invest.*, **72**, 1844.

PALMER, W.L. & HUMPHREYS, E.M. (1944). Gastric carcinoma: observations on peptic ulceration and healing. *Gastroenterology*, **3**, 257.

SAITO, T., INOKUCHI, K., TAKAYAMA, S. & SUGIMURA, T. (1970). Sequential morphological changes in *N*-methyl-*N'*-nitro-*N*-nitrosoguanidine carcinogenesis in the glandular stomach of rats. *J. Natl Cancer Inst.*, **44**, 769.

SEITZ, H.K., CZYGAN, P., KIENAPFEL, H. and 3 others (1983). Changes in gastrointestinal DNA synthesis produced by acute and chronic ethanol consumption in the rat: a biochemical study. *Z. Gastroenterol.*, **21**, 79.

SHAY, H., SUN, D.C.H. & GRUENSTEIN, M. (1954). A quantitative method for measuring spontaneous gastric secretion in the rat. *Gastroenterology*, **26**, 906.

SIEGEL, S. (1956). *Nonparametric Statistics for the Behavioral Sciences*. McGraw-Hill: New York.

SNEDECOR, C.W. & COCHRAN, W.G. (1967). *Statistical Methods*. Iowa University Press: Ames, Iowa.

STEFANINI, M., DEMARTINO, C. & ZAMBONI, L. (1967). Fixation of ejaculated spermatozoa for electron microscopy. *Nature*, **216**, 173.

TADA, T., KODAMA, T., WATANABE, S., SATO, Y. & SHIMOSATO, Y. (1985). Cell kinetics studies by the use of anti-bromodeoxyuridine monoclonal antibody and their clinical application. *Igaku-no-ayumi*, **135**, 510.

TAKAHASHI, M., HASEGAWA, R., FURUKAWA, F. and 3 others (1986). Effects of ethanol, potassium metabisulfite, formaldehyde and hydrogen peroxide on gastric carcinogenesis in rats after initiation with *N*-methyl-*N'*-nitro-*N*-nitrosoguanidine. *Jpn. J. Cancer Res.*, **77**, 118.

TATSUTA, M., IISHI, H., YAMAMURA, H. and 3 others (1988). Effect of cimetidine on inhibition by tetragastrin of carcinogenesis induced by *N*-methyl-*N'*-nitro-*N*-nitrosoguanidine in Wistar rats. *Cancer Res.*, **48**, 1591.

WILLEMS, G., VANSTEENKISTE, Y. & SMETS, P.H. (1971). Effects of ethanol on the cell proliferation kinetics in the fundic mucosa of dogs. *Am. J. Dig. Dis.*, **16**, 1057.

WILLIAMS, A.W. (1956). Effects of alcohol on gastric mucosa. *Br. Med. J.*, **i**, 256.

WILLIAMS, R.R. & HORM, J.W. (1977). Association of cancer sites with tobacco and alcohol consumption and socioeconomic status of patients: interview study from the third national cancer survey. *J. Natl Cancer Inst.*, **58**, 525.

WORLD HEALTH ORGANIZATION (1964). Cancer agents that surround us. *World Health.*, **9**, 16.